# Macrophyte diversity in rivers and streams of the Vologda Region and several other regions of Russia

**DOI:** 10.3897/BDJ.9.e76947

**Published:** 2021-12-03

**Authors:** Dmitriy A. Philippov, Aleksandra S. Komarova

**Affiliations:** 1 Papanin Institute for Biology of Inland Waters Russian Academy of Sciences, Borok, Russia Papanin Institute for Biology of Inland Waters Russian Academy of Sciences Borok Russia; 2 Tyumen State University, AquaBioSafe, Tyumen, Russia Tyumen State University, AquaBioSafe Tyumen Russia

**Keywords:** Russia, Eastern Europe, Western Siberia, dataset, macrophytes occurrences, flora of rivers, flora of streams, data paper, rare species

## Abstract

**Background:**

The data paper contains the authors’ materials on the diversity of macrophytes, macroscopic plants regardless of their taxonomic position, in rivers and streams of East European Russia and Western Siberia. These data, collected on 247 rivers and 32 streams in 13 administrative regions of the Russian Federation, were provided as an occurrence dataset presented in the form of GBIF-mediated data. The main portion of the data was obtained in water objects of the Vologda Region (5201 occurrences). In addition, occurrences from the Arkhangelsk Region (347 occurrences), Khanty-Mansi Autonomous Okrug (159), Yaroslavl Region (132), Novgorod Region (97), Kostroma Region (41), Republic of Karelia (31), Sverdlovsk Region (29), Komi Republic (28), Orenburg Region (26), Chelyabinsk Region (22), Voronezh Region (22) and Tyumen Region (18) were given. The studies were carried out mainly in the southern and middle taiga and, to a lesser extent, in the northern taiga and the forest-steppe. The analysed watercourses belong to five drainage basins: the Azov Sea, the Baltic Sea, the White Sea, the Caspian Sea and the Kara Sea. The dataset contains materials on the diversity of Plantae (6094 occurrences) and Chromista (59 occurrences). This paper, in a standardised form, summarises mostly unpublished materials on the biodiversity of lotic ecosystems.

**New information:**

The paper summarises the data obtained in long-term studies of phytodiversity in a range of rivers and streams of East European Russia and, fragmentarily, Western Siberia. A total of 6153 occurrences were included in the dataset. According to the GBIF taxonomic backbone, the dataset comprises 292 taxa, including 280 lower-rank taxa (species, subspecies, varieties) and 12 taxa identified to the genus level. All the occurrences are published openly through the Global Biodiversity Information Facility (GBIF) for the first time. Most of the data were stored in field diaries and, thus, by adding the data in GBIF, we believe that other researchers could benefit from it.

## Introduction

Macrophytes are an essential component of lotic ecosystems, especially in terms of biodiversity (Chambers et al. 2008), playing an important functional role, providing trophic resources and habitat for other aquatic organisms (Biggs 1996, Grenouillet et al. 2002) and regulating water flows and their chemistry (e.g. Carpenter and Lodge 1986, Clarke and Wharton 2001, Horvath 2004, Gurnell et al. 2006). Under the Water Framework Directive (European Commision 2000), macrophytes are one of the key indicators used for environmental health assessment.

The study summarises the authors’ materials collected in the rivers of the European part of Russia between 2005 and 2021 and Western Siberia in 2021. A small part (5.5%) of the data was published in the research articles (Ivicheva et al. 2018, Chernova and Philippov 2019, Chernova et al. 2019), but mainly as short notes on the new records (Dulin et al. 2009, Afonina et al. 2010, Dulin and Philippov 2010, Abolin et al. 2011, Bobrov and Philippov 2012, Sofronova et al. 2012, Chemeris and Bobrov 2013, Chemeris et al. 2013, Shabunov and Philippov 2014, Sofronova et al. 2015, Vishnyakov and Philippov 2018, Vishnyakov et al. 2020, Vishnyakov et al. 2021).

All the raw data on the biodiversity in rivers and streams are provided in an occurrence dataset (Philippov and Komarova 2021).

## Project description

### Title

Diversity, distribution, ecology, biology of aquatic and semi-aquatic plants in the European North

### Personnel

Dmitriy A. Philippov

## Sampling methods

### Study extent

A list of records of macrophytes in rivers and streams of European Russia and Western Siberia is presented. By macrophytes, we understood macroscopic plants, regardless of their taxonomic position and ecological characteristics. Macrophytes include vascular plants, mosses, liverworts and large multicellular algae (Papchenkov et al. 2003). We determined the flora of rivers and streams as aquatic species and species directly related to the aquatic environment (helophytes, plants of the water’s edge, amphibious plants, hygrophytes, plants of drying sandbanks).

### Sampling description

Field studies were carried out from May to October, mainly during the greatest development of macrophytes (July and August). The composition of the flora of rivers and streams was established during route field studies. We studied all available microhabitats in the channel and coastal parts of water bodies, including those differing in current velocity, sediments, depths and macrophyte canopy development. When studying streams, the route, as a rule, ranged from 50 to 150 m; on rivers, usually from 100 to 1000 m. In the Vologda Region, studies were carried out in all landscapes; several objects per type of landscape were selected depending on the density of the river network. In other regions, studies were carried out along with the study of other wetland ecosystems. In all studied regions, one route per water object was made. In the field, photographs of plants and their communities were taken, floristic lists were compiled and the main hydrochemical parameters were measured (water temperature, pH and TDS). The studies of the macrophyte community composition were conducted both purposefully and along with the studies of other groups of aquatic organisms. Macrophyte samples were collected; they are currently stored and being registered in the Herbaria of Mire Research Group, Papanin Institute for Biology of Inland Waters Russian Academy of Sciences (coded MIRE) and Vologda State University (coded VO). More than 1000 macrophyte specimens were collected in total.

### Quality control

The data were collected and identified by scientists from the Papanin Institute for Biology of Inland Waters Russian Academy of Sciences (IBIW RAS). The accuracy of determination of some samples was confirmed by experts from the Institute of Biology of Komi Scientific Centre of the Ural Branch of the Russian Academy of Sciences and Institute of Biology of Karelian Research Centre of the Russian Academy of Sciences

### Step description

Research problem formulation.

Logistic issues resolution, including the choice of routes, water objects, time and duration of work.

Field stage: obtaining samples and other original materials on the flora of rivers and streams. In the field, pictures of plants and floristic lists were taken, some species were collected in a herbarium; several hydrochemical parameters (water temperature, total dissolved solids, pH and electrical conductivity) were measured using portable devices (Philippov et al. 2017).

Data collection: analysis of samples not identified in the field or verification of the identification data by the experts. The keys by Tzvelev (2000), Ignatov and Ignatova (2003), Ignatov and Ignatova (2004), Lisitsyna et al. (2009) and Maevskii (2014) were used in the study. Herbarium materials were transferred for processing to the Herbarium of the Mire Research Group of Papanin Institute for Biology of Inland Waters Russian Academy of Sciences (MIRE).

Records list compilation. The dataset field names were chosen according to Darwin Core (Wieczorek et al. 2012). Georeferencing was made by fixing the coordinates of the middle part of the studied river site using a GPS navigator or Google maps. Coordinates accuracy was set to the fourth digit. In all cases, the WGS-84 coordinate system was used.

## Geographic coverage

### Description

**Figure 1.**
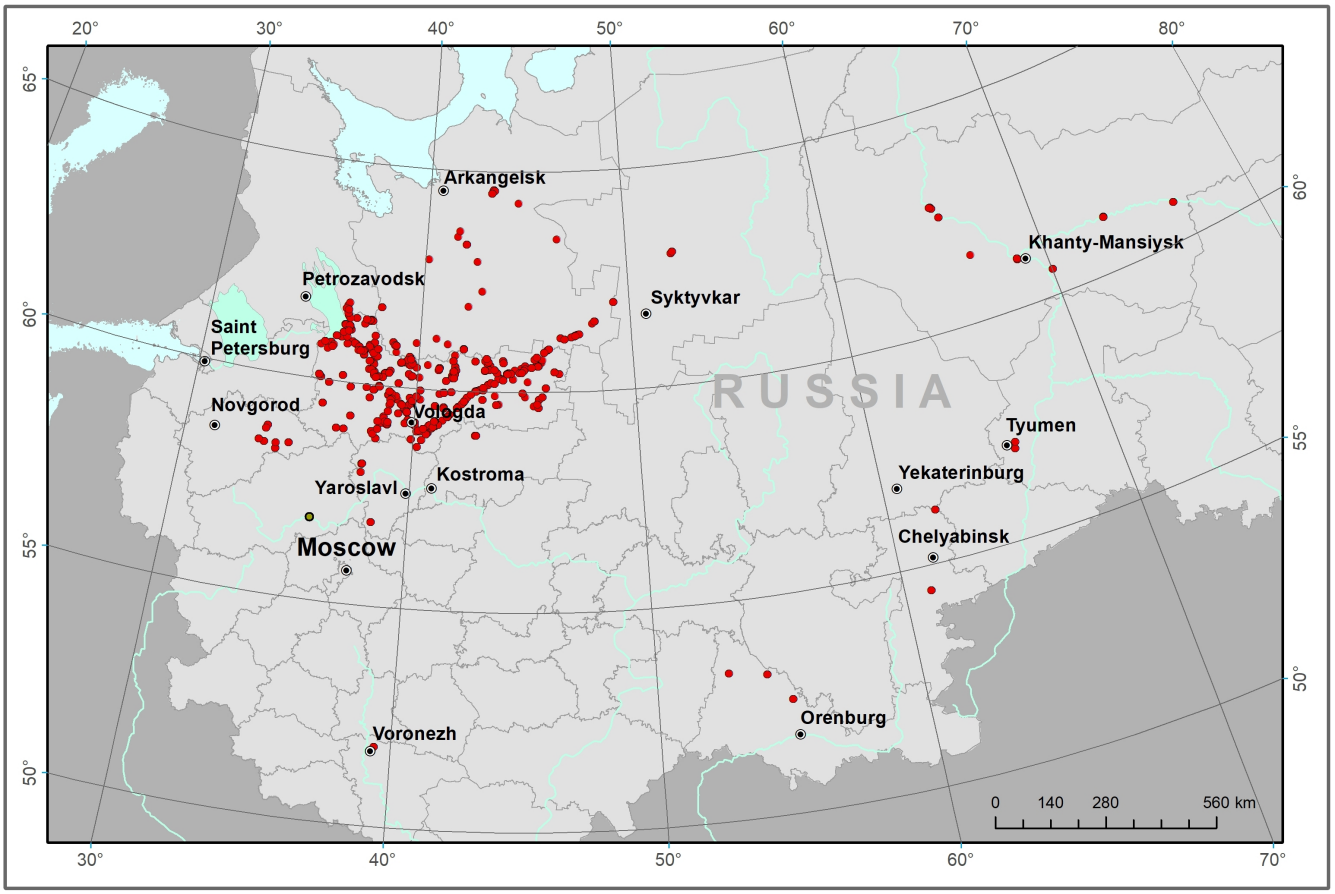
Study area and occurrences localities (red circles). The map was constructed using ArcGis 10 software.

The studies were carried out in various parts of European Russian and Western Siberia, mainly in the southern and middle taiga and a lesser number in the northern taiga and the forest-steppe. The analysed watercourses belong to five drainage basins: the Azov Sea, the Baltic Sea, the White Sea, the Caspian Sea and the Kara Sea. The coordinates of the northernmost site were 64.5729N, 43.2959E, the southernmost site 51.8139 N, 39.3836 E, the westernmost site 58.4353N, 33.2828E and the easternmost site 60.8691N, 76.4263E (Fig. 1).

**Figure 2.**
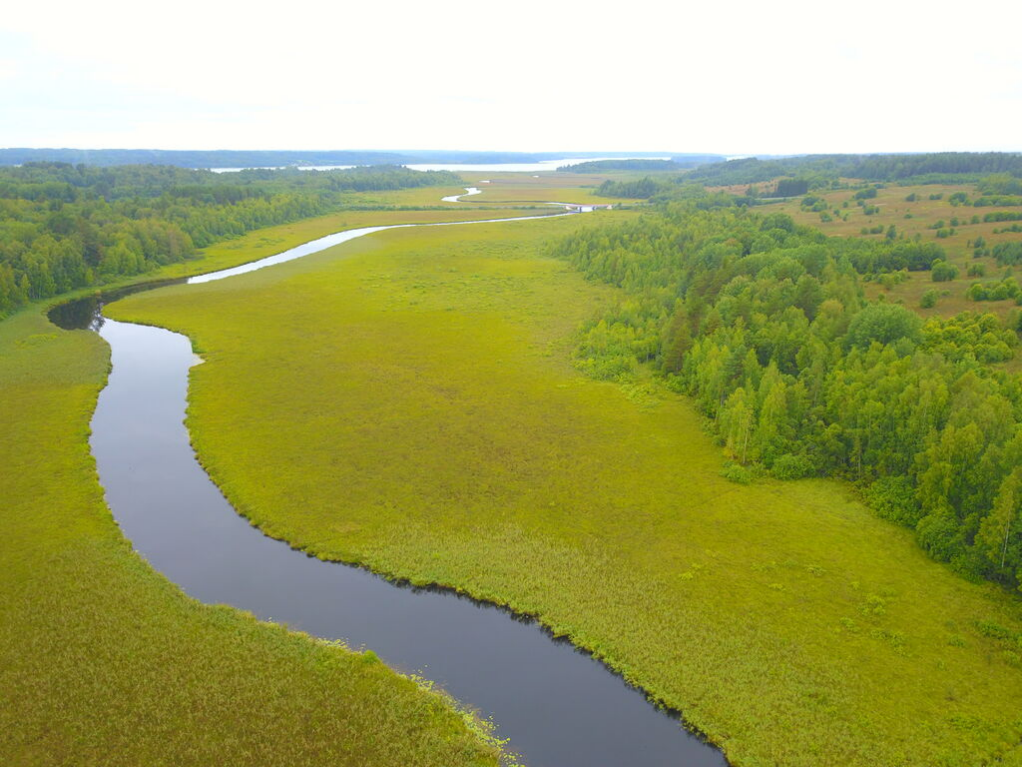
Ileksa River passing through the eutrophic mire (Vologda Region, Russia). Photo by Dmitriy A. Philippov (2019).

**Figure 3.**
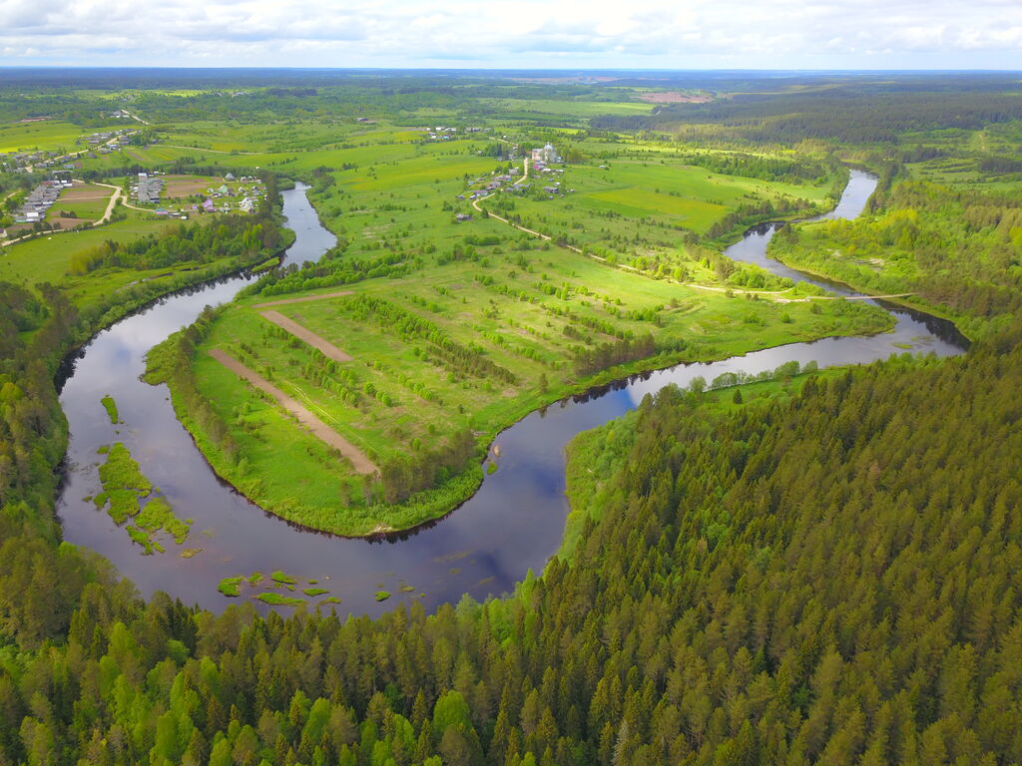
Vaga River flowing in upland (Vologda Region, Russia). Photo by Dmitriy A. Philippov (2021).

**Figure 4.**
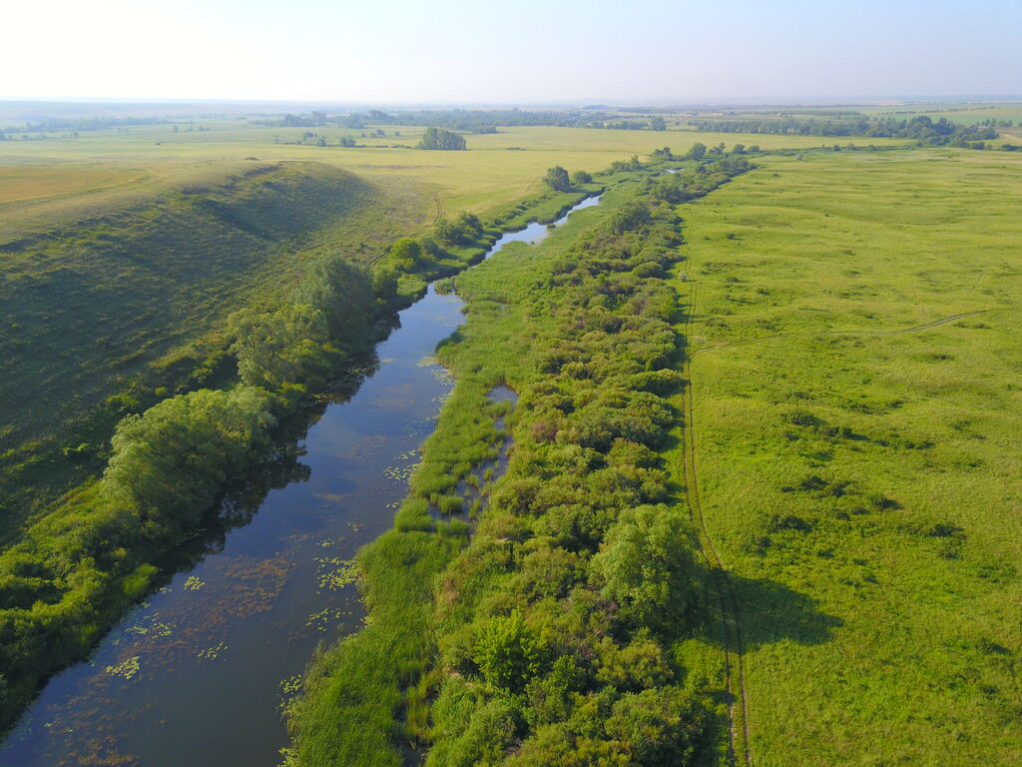
Salmysh River flowing in the forest-steppe (Orenburg Region, Russia). Photo by Dmitriy A. Philippov (2021).

**Figure 5.**
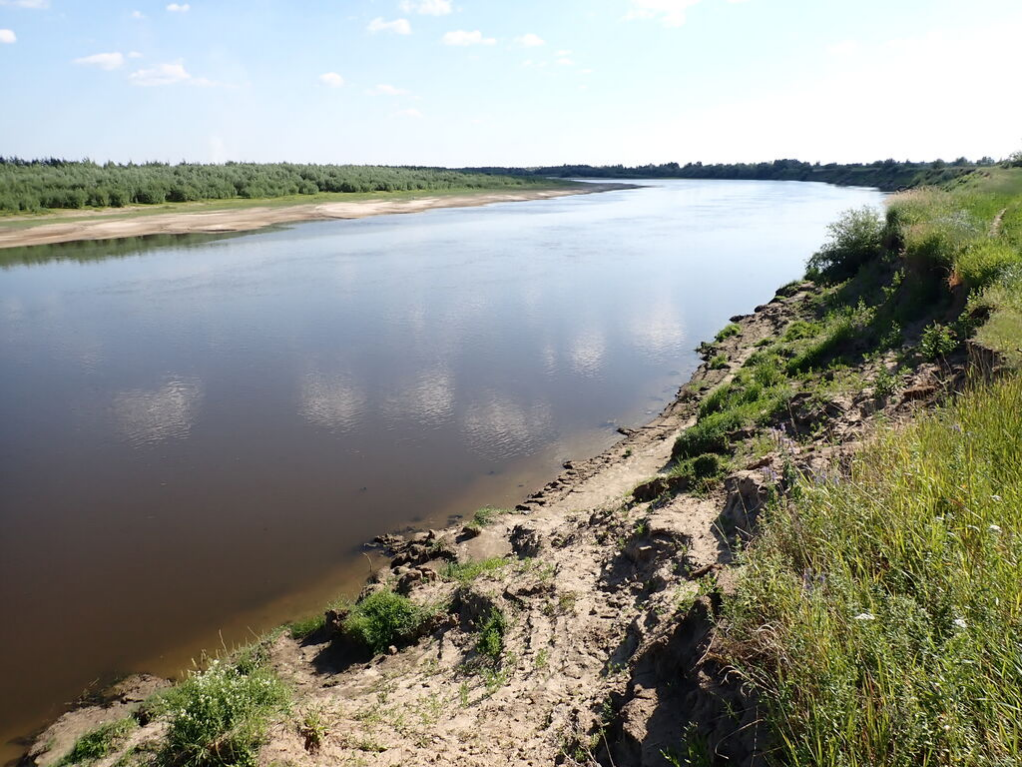
Тura River flowing in the West Siberian Plain (Tyumen Region, Russia). Photo by Dmitriy A. Philippov (2021).

**Figure 6.**
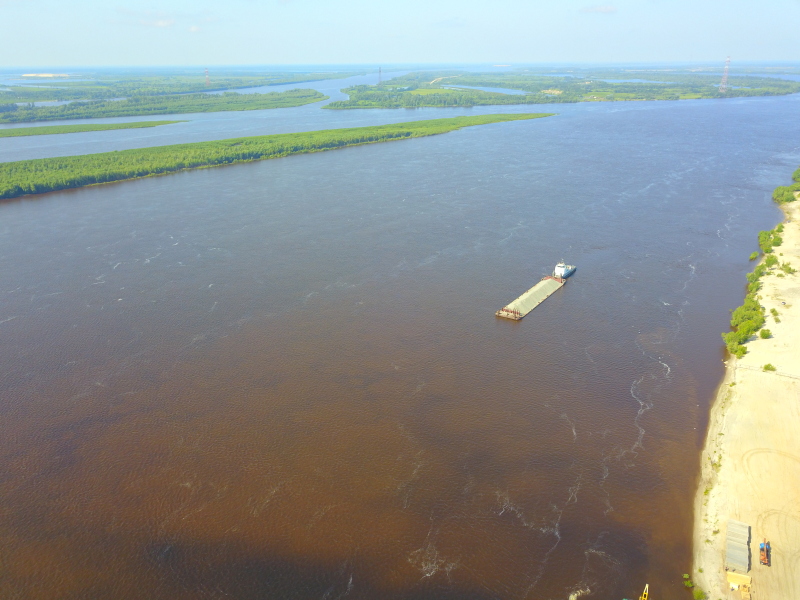
Ob River in Surgut City (Khanty-Mansi Autonomous Okrug, Russia). Photo by Dmitriy A. Philippov (2021).

Some examples of the studied rivers are given below (Figs 2, 3, 4, 5, 6).

### Coordinates

51.814 and 64.573 Latitude; 33.283 and 76.426 Longitude.

## Taxonomic coverage

### Description

The dataset contains 292 taxa of Tracheophyta, Bryophyta, Marchantiophyta, Chlorophyta, Charophyta, Ochrophyta, Rhodophyta, including 280 lower-rank taxa (species, subspecies, varieties) and 12 taxa identified to the genus level.

### Taxa included

**Table:** 

Rank	Scientific Name	
kingdom	Chromista	
phylum	Ochrophyta	
kingdom	Plantae	
phylum	Tracheophyta	
phylum	Marchantiophyta	
phylum	Bryophyta	
phylum	Chlorophyta	
phylum	Charophyta	
kingdom	Rhodophyta	

## Traits coverage

### Data coverage of traits

PLEASE FILL IN TRAIT INFORMATION HERE

## Temporal coverage

### Notes

2005 to 2021

## Usage licence

### Usage licence

Other

### IP rights notes

This work is licensed under a Creative Commons Attribution (CC-BY) 4.0 Licence.

## Data resources

### Data package title

Data on macrophyte diversity in rivers and streams of the Vologda Region and several other regions of Russia.

### Resource link

https://www.gbif.org/dataset/1c52ce65-b940-4bb8-8666-3025e58ef9ed

### Alternative identifiers

http://gbif.ru:8080/ipt/resource?r=rivers_and_streams

### Number of data sets

1

### Data set 1.

#### Data set name

Data on macrophyte diversity in rivers and streams of the Vologda Region and several other regions of Russia.

#### Data format

Darwin Core

#### Number of columns

31

#### Character set

Occurrence dataset

#### Download URL

https://www.gbif.org/dataset/1c52ce65-b940-4bb8-8666-3025e58ef9ed

#### Data format version

1.1

#### Description

The dataset contains the authors’ materials on macrophyte diversity (macroscopic plants regardless of their taxonomic position) in rivers and streams of East European Russia and Western Siberia. Overall, the dataset contains materials on Plantae (6094 occurrences: Tracheophyta - 5506, Marchantiophyta - 233, Bryophyta - 196, Chlorophyta - 94, Charophyta - 49, Rhodophyta - 16) and Chromista (59 occurrences: Ochrophyta - 59) diversity. A total of 6153 occurrences (280 lower-rank taxa and 12 taxa identified to the genus level) are included in the dataset.

**Data set 1.** 

Column label	Column description
occurrenceID	An identifier for the record, unique within this dataset. An abbreviation in the identifier' number (MiReGr_RiverPhytoDiv_xxxxx).
basisOfRecord	The specific nature of the data record in standard label of one of the Darwin Core. A constant ("HumanObservation").
scientificName	The full scientific name, with authorship and date information, if known.
eventDate	The date when the occurrence was recorded.
taxonRank	The taxonomic rank.
kingdom	The full scientific name of the kingdom in which the taxon is classified.
phylum	The full scientific name of the phylum or division in which the taxon is classified.
class	The full scientific name of the class in which the taxon is classified.
order	The full scientific name of the order in which the taxon is classified.
family	The full scientific name of the family in which the taxon is classified.
genus	The full scientific name of the genus in which the taxon is classified.
habitat	A category or description of the habitat in which the occurrence was recorded (river or stream).
decimalLatitude	The geographic latitude in decimal degrees of the geographic centre of the data sampling place.
decimalLongitude	The geographic longitude in decimal degrees of the geographic centre of the data sampling place.
geodeticDatum	The ellipsoid, geodetic datum or spatial reference system (SRS) upon which the geographic coordinates given in decimalLatitude and decimalLongitude are based. A constant ("WGS84").
coordinateUncertaintyInMetres	The maximum uncertainty distance in metres (30-500 m range).
coordinatePrecision	A decimal representation of the precision of the coordinates given in the decimalLatitude and decimalLongitude. A constant ("0.0001").
countryCode	The standard code for the Russian Federation according to ISO 3166-1-alpha-2 (RU).
country	Country name (Russian Federation).
stateProvince	The name of the next smaller administrative region than country ("oblast", "avtonomniy okrug", "respublica", "kray", "region") in which the location occurs. A variable (for example, "Vologda Region").
county	District (‘rayon’, in Russian administrative subdivision system) name. The second-level administrative division.
locality	The specific description of the place. This term may contain information modified from the original to correct perceived errors or standardise the description. A variable (names of rivers or springs).
year	The four-digit year in which the Event occurred, according to the Common Era Calendar.
month	The integer month in which the Event occurred.
day	The integer day of the month on which the Event occurred.
recordedBy	List of persons who collected field data.
identifiedBy	A person who assigned the Taxon to the subject.
dateIdentified	The date when the taxonomic identification happened.
associatedReferences	List of literature references associated with the occurrences (articles).
acceptedNameUsage	The full name, with authorship and date information, if known, of accepted taxon.
taxonomicStatus	The taxonomic status of a taxon. A variable (accepted or synonym).

## Additional information

The data were collected on 247 rivers and 32 streams of East European Russia and Western Siberia and included 6153 occurrences of macrophytes (280 lower-rank taxa and 12 taxa identified to the genus level).

**Figure 7.**
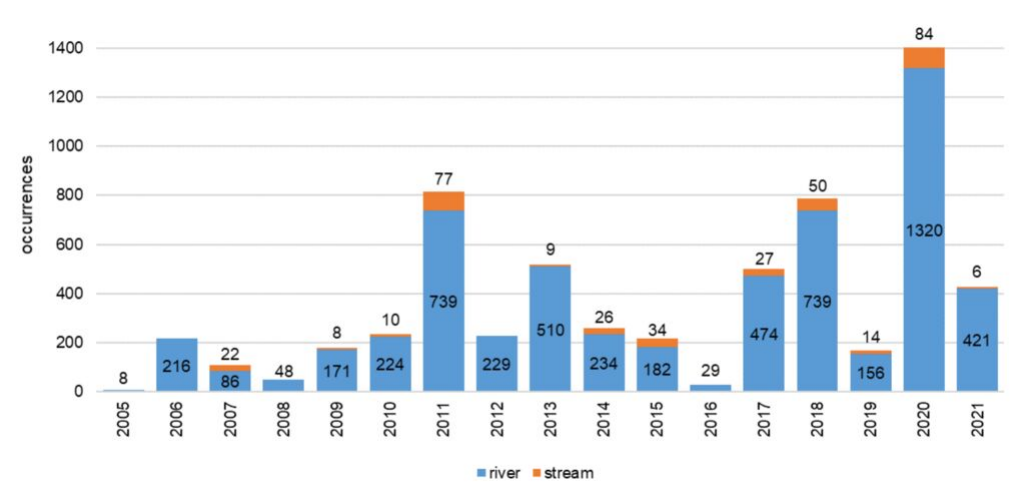
Distribution of macrophyte occurrences in rivers and streams from 2005 to 2021.

These studies have been conducted since 2005. From 8 to 1404 occurrences were registered each year (Fig. 7). The most productive years in respect to macrophyte studies in rivers and streams were 2011, 2013, 2017, 2018, 2020 and 2021. In any given year, the greatest emphasis was on rivers, not streams.

The studies were conducted in 13 administrative regions of Russia (Table 1). The greatest number of water objects were investigated in the Vologda Region (190 rivers and 26 streams). In the Arkhangelsk Region, 30 rivers and streams were studied; in other 11 administrative regions, from 1 to 7 rivers and streams. Therefore, the most occurrences and lower-rank taxa records came from the Vologda Region (5201 and 266, respectively).

On average, 22 ± 1.4 occurrences (from 1 to 176) came from each given water object. Almost half of all occurrences (48.9%) were registered on 50 rivers (Table 2).

The studies have provided data on the locations of a number of rare macrophyte species. For example, in the Vologda Region, 32 species listed in the Red Data Book of the Vologda Region (Suslova et al. 2013, Anonymous 2015) were registered, including 15 protected species: Critically Endangered (CR) - *Batrachiumpenicillatum*, Endangered (EN) - *Potamogetoncrispus*, Vulnerable (VU) - *Batrachiumcircinatum*, *Carexatherodes*, Carexelatasubsp.omskiana (Meinsh.) Jalas [as *C.omskiana* (Meinsh.) Jalas], *Ricciacanaliculata*, Near Threatened (NT) - *Aegagropilalinnaei*, *Moliniacoerulea*, *Tolypellaprolifera*, Least Concern (LC) - *Carexpseudocyperus*, *Charavirgata*, *Equisetumvariegatum*, *Hygroamblystegiumfluviatile*, *Nitellasyncarpa*, Seneciopaludosussubsp.lanatus [as *S.tataricus* Less.] and 17 species assigned with the status “biological control required”: *Batrachiumeradicatum*, *Batrachiumtrichophyllum*, *Charatomentosa*, *Eleocharisuniglumis*, *Humuluslupulus*, *Hydrocharismorsus-ranae*, *Irispseudacorus*, *Nymphaeacandida*, *Potamogetonberchtoldii*, *Potamogetonfiliformis*, *Rumexhydrolapathum*, *Scapaniasubalpina*, *Scolochloafestucacea*, *Sparganiumnatans*, *Stratiotesaloides*, *Typhaangustifolia* and *Utriculariaintermedia*.

## Figures and Tables

**Table 1.** Number of occurrences and taxa of macrophytes in rivers and streams in the studied regions.

**Region**	**Number of rivers and streams**	**Number of occurrences**	**Number of lower-rank taxa**
Arkhangelsk Region	30	347	116
Chelyabinsk Region	1	22	22
Khanty-Mansi Autonomous Okrug	5	159	53
Komi Republic	3	28	14
Kostroma Region	1	41	30
Novgorod Region	7	97	50
Orenburg Region	3	26	23
Republic of Karelia	7	31	15
Sverdlovsk Region	7	29	29
Tyumen Region	2	18	17
Vologda Region	216	5201	266
Voronezh Region	1	22	22
Yaroslavl Region	4	132	61
**All regions**	**287**	**6153**	**280**

**Table 2.** Top 50 rivers by the number of macrophyte occurrences amongst the 279 studied rivers and streams of Russia.

**River**	**Region**	**Number of occurrences**
Syamzhena River	Vologda Region	176
Kostyuga-1 River	Vologda Region	138
Indomanka River	Vologda Region	119
Vologda River	Vologda Region	118
Kema River	Vologda Region	110
Ob River	Khanty-Mansi Autonomous Okrug	108
Svid' River	Arkhangelsk Region	99
Soyda River	Vologda Region	95
Andoma River	Vologda Region	88
Vozhega River	Vologda Region	88
Vaga River	Vologda Region	76
Il'd River	Yaroslavl Region	74
Bol'shoy Pungul River	Vologda Region	72
Ileksa River	Vologda Region	67
Uftyuga River (= tarnogskaya Uftyuga River)	Vologda Region	67
Ukhtomka River	Vologda Region	67
Votcha River	Vologda Region	63
Iksoma River	Vologda Region	57
Nyuksha River	Vologda Region	55
Tagazhma River	Vologda Region	54
Palaya River	Vologda Region	53
Sora River	Vologda Region	53
Koloshma River	Vologda Region	50
Uftyuga River (= nyuksenskaya Uftyuga River)	Vologda Region	50
Chernyy Shingar' River	Vologda Region	49
Kubena River	Vologda Region	49
Bol'shaya Salanga River	Vologda Region	47
Povreka River	Vologda Region	45
Il'chuga River	Vologda Region	44
Kokshen'ga River	Vologda Region	42
Kikht' River	Vologda Region	42
Komyola River	Vologda Region	41
Uskala River	Vologda Region	41
Vocha River	Kostroma Region	41
Pes'ya Den'ga River	Vologda Region	40
Strel'na River	Vologda Region	40
Sheybukhta River	Vologda Region	38
Pinega River	Arkhangelsk Region	37
Pocha River	Vologda Region	37
Megra River	Vologda Region	36
Ukhtomitsa River (= kirillovskaya Ukhtomitsa River)	Vologda Region	36
Bol'shaya Runga River	Vologda Region	35
Chuzhga River	Vologda Region	35
Kiuy River	Vologda Region	35
Okhotka River	Vologda Region	35
Nerel' River	Yaroslavl Region	34
Suda River	Vologda Region	34
Chundruchey River	Vologda Region	33
Kovzha River	Vologda Region	33
Yema River	Vologda Region	33
**Total**		**3009**

## References

[B7510672] Abolin A. A., Andreeva E. N., Afonina O. M., Badmaeva N. K., Bakalin V. A., Belkina O. A., Borovichev E. A., Chemeris E. V., Cherdantseva V. Y., Cherednichenko O. V., Czernyadjeva I. V., Doroshina G. Y., Dulin M. V., Ibatullin A. A., Ignatov M. S., Ignatova E. A., Kokoshnikova Y. S., Konstantinova N. A., Kotseruba V. V., Malashkina E. V., Mamontov Y. S., Notov A. A., Opmanis A. G., Philippov D. A., Potemkin A. D., Reriha I. S., Shestakova A. A., Shilnikov D. S., Sofronova E. V., Susko U. A., Teleganova V. V., Tubanova D. Y. (2011). New records. Arctoa.

[B7510770] Afonina O. M., Akatova T. V., Baisheva E. Z., Belkina O. A., Bezgodov A. G., Borovichev E. A., Czernyadjeva I. V., Boychuk M. A., Doroshina G. Y., Dulin M. V., Fedosov V. E., Ignatov M. S., Ignatova E. A., Konstantinova N. A., Krivobokov L. V., Kučera J., Kushnevskaja E. V., Maksimov A. I., Maksimova T. A., Mamontov Y. S., Notov A. A., Philippov D. A., Potemkin A. D., Ryazanova D. T., Schilnikov D. S., Savchenko A. N., Sofronov R. R., Sofronova E. V., Tubanova D. Y., Urbanavichene I. N., Urbanavichyus G. P., Volkova E. M., Zheleznova G. V. (2010). New records. Arctoa.

[B7517782] Anonymous (2015). Resolution of the Government of Vologda Region from 24.02.2015 №125 «On approval of list of rare and endangered species (intraspecific taxa) plants and fungi, which feature in the Red Data Book of Vologda Region». https://vologda-oblast.ru/dokumenty/393552/.

[B7517427] Biggs B. J. (1996). Hydraulic habitat of plants in streams. River Research and Applications.

[B7510604] Bobrov A. A., Philippov D. A. (2012). *Myriophyllumsibiricum* (Haloragaceae) in Vologda region. Vestnik of Saint Petersburg University. Biology.

[B7518107] Carpenter S. R., Lodge D. M. (1986). Effects of submersed macrophytes on ecosystem processes. Aquatic Botany.

[B7517382] Chambers P. A., Lacoul P., Murphy K. J., Thomaz S. M. (2008). Global diversity of aquatic macrophytes in freshwater. Hydrobiologia.

[B7510652] Chemeris E. V., Bobrov A. A. (2013). *Aegagropilalinnaei* (Cladophoraceae, Chlorophyta) in rivers of the northern European Russia. Botanicheskii Zhurnal.

[B7510662] Chemeris E. V., Bobrov A. A., Philippov D. A. (2013). Stoneworts (Charophyta) of watercourses in Vologda region. Vestnik of Saint Petersburg University. Biology.

[B7510584] Chernova A. M., Philippov D. A. (2019). Stocks of *Nupharlutea* (L.) Sm. in small river Ild (Yaroslavl Region, Russia). Field Biologist Journal.

[B7523787] Chernova A. M., Czhobadze A. B., Levashov A. N., Philippov D. A. (2019). Flora of waterbodies of the Volga River Basin: additions and updates on the Vologda Region, Russia. Samarskaya Luka: problems of regional and global ecology.

[B7517391] Clarke S. J., Wharton G (2001). Sediment nutrient characteristics and aquatic macrophytes in lowland English rivers. The Science of the Total Environment.

[B7510556] Dulin M. V., Philippov D. A., Karmazina E. V. (2009). Current state of knowledge of the liverwort and hornwort flora of the Vologda Region, Russia. Folia Cryptogamica Estonica.

[B7510594] Dulin M. V., Philippov D. A. (2010). Additions to the liverworts flora of the Vologda Region. Herald of Tver State University. Series: Biology and Ecology.

[B7517437] European Commision (2000). Directive 2000/60/EC of the European Parliament and of the Council of 23 October 2000 establishing a framework for the community action in the field of water policy. Official Journal of the European Commission - Legis.

[B7517400] Grenouillet G., Pont D., Seip K. L. (2002). Abundance and species richness as a function of food resources and vegetation structure: juvenile fish assemblages in rivers. Ecography.

[B7517409] Gurnell A. M., van Oosterhout M. P., de Vlieger B., Goodson J. M. (2006). Reach-scale interactions between aquatic plants and physical habitat: River Frome, Dorset. River Research and Applications.

[B7517418] Horvath T. G. (2004). Retention of particulate matter by macrophytes in a first-order stream. Aquatic Botany.

[B7510011] Ignatov M. S., Ignatova E. A. (2003). Moss flora of the Middle European Russia. Vol. 1: Sphagnaceae – Hedwigiaceae. Arctoa.

[B7510029] Ignatov M. S., Ignatova E. A. (2004). Moss flora of the Middle European Russia. Vol. 2: Fontinalaceae – Amblystegiaceae. Arctoa.

[B7510546] Ivicheva K. N., Makarenkova N. N., Zaytseva V. L., Philippov D. A. (2018). Influence of flow velocity, river size, a dam, and an urbanized area on biodiversity of lowland rivers. Biosystems Diversity.

[B7510128] Lisitsyna L. I., Papchenkov V. G., Artemenko V. I. (2009). Флора водоёмов волжского бассейна. Определитель сосудистых растений.

[B7510138] Maevskii P. F. (2014). Флора средней полосы европейской части России.

[B7510147] Papchenkov V. G., Scherbakov A. V., Lapirov A. G. (2003). Основные гидроботанические понятия и сопутствующие им термины: Проект.

[B7510196] Philippov D. A., Prokin A. A., Przhiboro A. A. (2017). Методы и методики гидробиологического исследования болот: учебное пособие.

[B7515119] Philippov D. A., Komarova A. S. (2021). Data on macrophyte diversity in rivers and streams of the Vologda Region and several other regions of Russia. https://www.gbif.org/dataset/1c52ce65-b940-4bb8-8666-3025e58ef9ed.

[B7510574] Shabunov A. A., Philippov D. A. (2014). Rare vascular plants and vertebrate animals findings in the southern part of Gryazovets district: additions to Vologda Region Red book. Vestnik of Kostroma State University.

[B7510936] Sofronova E. V., Abakarova A. S., Afonina O. M., Badmaeva N. K., Borovichev E. A., Boychuk M. A., Czernyadjeva I. V., Doroshina G. Y., Dulin M. V., Dyachenko A. P., Fedosov V. E., Ibatullin A. A., Ignatov M. S., Ignatova E. A., Ivanova E. I., Ivchenko T. G., Kokoshnikova Y. S., Kozhin M. N., Kuzmina E. Y., Maksimov A. I., Maksimova T. A., Malashkina E. V., Mamontov Y. S., Moshkovskii S. A., Notov A. A., Philippov D. A., Potemkin A. D., Pisarenko O. Y., Preobrazhenskaja E. S., Shafigullina N. R., Taran G. S., Teleganova V. V., Teplov K. Y., Terentyeva E. V., Tubanova D. Y., Zheleznova G. V. (2012). New bryophyte records. 1. Arctoa.

[B7510877] Sofronova E. V., Abdurachmanova Z. I., Afonina O. M., Akatova T. V., Andrejeva E. N., Bakalin V. A., Bezgodov A. G., Borovichev E. A., Czernyadjeva I. V., Doroshina G. Y., Dulin M. V., Fedosov V. E., Golovina E. O., Ignatov M. S., Ignatova E. A., Kotkova V. M., Kozhin M. N., Kučera J., Kurbatova L. E., Kushnevskaya E. V., Leushina E. G., Makarova M. A., Maksimova А. Y., Nikolajev I. A., Philippov D. A., Popova N. N., Potemkin A. D., Prelovskaya E. S., Teleganova V. V., Vilnet A. A., Volkova E. M., Zolotukhin N. I. (2015). New bryophyte records. 5. Arctoa.

[B7517771] Suslova T. A., Czhobadze A. B., Philippov D. A., Shiryaeva O. S., Levashov A. N. (2013). A second edition of the Red Data Book of the Vologda Region: revisions in the lists of protected and biological control required species of plants and fungi. Phytodiversity of Eastern Europe.

[B7510156] Tzvelev N. N. (2000). Определитель сосудистых растений Северо-Западной России (Ленинградская, Псковская и Новгородская области).

[B7510615] Vishnyakov V. S., Philippov D. A. (2018). New records of charophytes (Charales) from the Northern European Russia. Botanicheskii Zhurnal.

[B7510639] Vishnyakov V. S., Romanov R. E., Chemeris E. V., Kipriyanova L. M., Chernova A. M., Komarova A. S., Philippov D. A. (2020). New records of *Vaucheria* (Ochrophyta, Xanthophyceae) in Russia. Novosti Sistematiki Nizhshikh Rastenii.

[B7510625] Vishnyakov V. S., Romanov R. E., Komarova A. S., Belyakov E. A., Moseev D. S., Churakova E. Y., Czhobadze A. B., Philippov D. A. (2021). New charophyte records (Characeae) in European Russia. Botanicheskii Zhurnal.

[B7510398] Wieczorek J., Bloom D., Guralnick R., Blum S., Döring M., Giovanni R., Robertson T., Vieglais D. (2012). Darwin Core: An evolving community-developed biodiversity data standard. PLOS One.

